# Nerve-Stretching in Tetanus

**Published:** 1876-09

**Authors:** 


Nerve-Stretching in Tetanus.—Various surgeons have recently commended this proceeding. Dr. Verneuil has lately adopted the practice. He exposed the median nerve at the elbow, and the ulnar at the wrist, and stretched them, the patient recovering. Mr. Callender, of London, advocates the trial of this method. In view of the unsatisfactory results of the treatment of traumatic tetanus, there is full justification for the performance of the operation, as at least a last resource, although he thinks it ought to be done, as in Verneuil's case, as soon as the signs of the disease are distinctly recognized.—*Med. and Surg. Reporter.*

